# Association of the eNOS E298D polymorphism and the risk of myocardial infarction in the Greek population

**DOI:** 10.1186/1471-2350-11-133

**Published:** 2010-09-20

**Authors:** Chaido Dafni, Nikolaos Drakoulis, Olfert Landt, Dimitris Panidis, Martin Reczko, Dennis V Cokkinos

**Affiliations:** 1 Department of Pharmaceutical Technology, School of Pharmacy, University of Athens, Panepistimiopolis Zografou, 15771, Athens, Greece; 2 TIB MOLBIOL, 22-23 Eresburstrasse, D-12103 Berlin, Germany; 3 Institute of Molecular Oncology, Biomedical Sciences Research Center "Alexander Fleming", P.O. Box 74145, 16602, Vari, Greece; 4 1st Cardiology Department, Onassis Cardiac Surgery Center, 356 Sygrou Avenue, 17674 Kallithea, Athens, Greece

## Abstract

**Background:**

Nitric oxide (NO), produced by endothelial nitric oxide synthase (eNOS), plays a key role in the regulation of vascular tone. Endothelium-derived NO exerts vasoprotective effects by suppressing platelet aggregation, leukocyte adhesion and smooth muscle cell proliferation. The E298D polymorphic variant of eNOS has been associated with myocardial infarction (MI), but data relating to this variant are divergent in Greece. Accordingly, we examined a possible association between the E298D polymorphism of the eNOS gene and MI in a subgroup of the Greek population.

**Methods:**

The study population consisted of 204 patients with a history of MI and 218 control subjects. All subjects were of Greek origin and were selected from the general population of the greater Athens area. Genotyping was performed with melting curve analysis (Lightcycler system) of polymerase chain reaction amplified products using hybridization probes.

**Results:**

According to the univariate findings, the risk for MI in E298D TT was 2.06 (95%CI: 1.06-4.00, p = 0.032) versus GG+GT and 2.34 (95%CI: 1.17-4.68, p = 0.016) versus GG. The risk for the T allele was estimated at 1.42 (95%CI, 1.06-1.89, p = 0.022) as compared to G allele. Regarding the additive model, one allele increase was associated with 43% higher risk of MI (OR = 1.43, 95%CI: 1.07-1.93, p = 0.018) as compared to the baseline category of homozygous GG. The positive association of TT versus GG+GT with MI risk remained even after adjusting for the main study covariates. Moreover, strong evidence was found for an increased risk for MI among carriers of the TT genotype who were smokers, hypertensive and had a family history of CAD.

**Conclusions:**

This study indicates that E298D polymorphism of the eNOS gene seems to be associated with MI occurrence in the Greek population. It is possible that TT genotype is closely linked to the etiology of MI even after adjusting for known MI risk factors.

## Background

Nitric oxide (NO), the endothelium-derived relaxing factor, is synthesized from L-arginine by at least three isoforms of the Nitric Oxide Synthase (NOS) (inducible NOS, iNOS or NOS2, constitutive neuronal NOS, nNOS or NOS1 and constitutive endothelial NOS, eNOS or NOS3). Nitric oxide, produced by eNOS, diffuses from the endothelium to vascular smooth muscle cells, where it increases the concentration of cyclic guanosine monophosphate (cGMP) by stimulating soluble guanylate cyclase, leading to vascular relaxation [1].

Endothelium-derived NO plays a key role in the regulation of vascular tone and displays vasoprotective effects by scavenging superoxide radicals [2] and suppressing platelet aggregation [3], leukocyte adhesion [4] and smooth muscle cell proliferation [5]. These actions suggest that endothelial NO may have an important atheroprotective role. Thus, an alteration in the activity of the vascular NO system could contribute to the pathogenesis of atherosclerosis and thrombosis.

The gene encoding eNOS is located on chromosome 7q35-36 and contains 26 exons that span 21 kb [6]. The common polymorphism G^894^T in exon 7 of the eNOS gene results in the substitution of glutamic acid (E) at codon 298 by aspartic acid (D) (E298D). This is the only known polymorphism changing the eNOS protein sequence, leading to speculation that genetic variation at this site may alter e-NOS activity or regulation and possibly leads to endothelial dysfunction and to pathogenesis of several cardiovascular diseases [7].

The 298D variant has been associated with myocardial infarction (MI) in Japanese [8,9], English [10], German [11] and American [12] populations, whereas other studies do not support these findings in Koreans [13,14] and in Caucasians, i.e. Austrian [15] French-Nothern Irish [16] and Dutch [17] populations. In Greece studies of the E298D polymorphism had controversial results [18-20]. Accordingly, it was the aim of the present study to investigate in a subgroup of the Greek population whether the E298D gene variation was related to the risk of myocardial infarction in the total population and among subjects who were at lower or higher risk of this disease.

## Methods

### Study population

A total of 422 subjects of Greek origin were prospectively enrolled in the study. Among them, 204 patients, diagnosed with an Acute Myocardial Infarction (AMI) or Non ST Segment Elevation Myocardial Infarction (NSTEMI) were compared to 218 control subjects.

The 204 patients with MI admitted to the 1st Department of Cardiology, Onassis Cardiac Surgery Center Athens, Greece from October 2007 to April 2008. Diagnosis of AMI and NSTEMI was made by chest symptoms, electrocardiographic changes and serum creatinine kinase-MB isoenzyme (CK-MB) elevations according to guidelines [21].

The control group consisted of 218 volunteers who were selected from the general population of the greater Athens area. Controls were not eligible to participate if they had a history of MI or angina, clinical evidence of coronary artery disease (CAD), stroke, or any atherosclerotic disease in the past, based on a detailed medical history and a physical examination followed by a normal electrocardiogram.

All subjects provided written informed consent and the study was approved by the hospital ethics committee. Subjects were evaluated with a detailed questionnaire that provided information on coronary risk factors, namely smoking, family history of CAD, hypertension, diabetes mellitus and hypercholesterolemia. Patients who used to smoke more than eight cigarettes per day for more than one year were considered to be smokers (current or ex-smokers). Body mass index (BMI) was calculated in all participants.

MI patients and controls were matched by gender and age ± five years. Controlling for basic socio-demographic characteristics in the statistical analysis, minimized further the consequences of potential confounding by those variables.

### Coronary risk factors

Hypertension was defined by a history of several blood pressure measurements with elevation of either systolic (> 140 mmHg) or diastolic (> 90 mmHg) blood pressure. Diabetes mellitus was defined by elevated blood glucose levels after fasting: (> 126 mg/dL) or 2 h after 75 g of oral glucose loading (> 200 mg/dL), or by anti-diabetic drug. Hypercholesterolemia was defined by elevated total serum cholesterol levels (> 200 mg/dL).

### DNA extraction and genotyping

Genomic DNA was extracted from peripheral blood leukocytes using standard methods (Micromix 660 DNA extraction kit, Talent, Italy). Purified DNA (concentration of 60 μg/ml) was stored at -20°C. The E298D polymorphism was determined by Real-Time Polymerase Chain Reaction analysis using the LightCycler 480 Instrument (Roche Diagnostics, Mannheim, Germany). The published probes [22] were found to fail to detect homozygous TT samples. Thus, novel oligonucleotide primers and probes were designed and validated by TIB-MOLBIOL, Berlin, Germany. The kit contains control DNA (human eNOS 298D mutant [mt], wild type [wt], heterozygous [hetero], 10^5 ^target equivalents per reaction). PCR primers 5'-AAggCAggAgACAgTggATggA and 5'-TgCTCCAggggCACCTCAAg and the probes CCAgATgATCCCCTAgAACTC--FL (T allele specific base underlined) and 640-CCTTCTgCCCCCCgAgCTggTCC--PH contained in the LightMix kit eNOS (TIB MOLBIOL, Berlin, Germany) were used in combination with the LightCycler 480 Genotyping Master and 96 well plates (Roche Diagnostics, Mannheim, Germany), following the instructions of the manufacturer. The tests were run with 5 μl (0.3 μg) sample DNA in a final volume of 20 μl; control DNA (human eNOS 298D [mt], [wt], [hetero]) was diluted 1:4. Each PCR run contained a negative (no template) control. To confirm reproducibility, mutation analysis of 9 random samples (3 wt/wt, 3 wt/mt and 3 mt/mt) was performed 10 times each.

The probes are designed to match with the T allele, thus reporting a lower melting temperature (Tm) for the wild type G allele. Distinction of wild type, mutant and heterozygous genotype can be easily accomplished by their respective melting temperatures.

After 10 min denaturation at 95°C the amplification was performed running 45 cycles 5 s at 95°C, 10 s at 60°C and 15 s at 72°C, following the instructions of the LightMix kit. The melting program included three steps: denaturalization at 95°C for 30 s, renaturation at 40°C for 1 min and then slowly raised to 80°C to allow monitoring of the decline of fluorescence generated by melting of the hybrids, as a function of temperature. Melting curves were automatically converted to fluorescence peaks with the LightCycler 480 Instrument analysis software, allowing the distinction of genotypes. The grouping software uses a curve shape-matching algorithm to identify wild type from mutant samples and cut-offs are based on variability from the wild type curve. The results were confirmed for homozygous mutant, homozygous wild type and heterozygous samples in all repeated measurements.

### Statistical analysis

Body mass index (BMI) was regarded as continuous variable, while age, gender, kind of genotype, and presence of all coronary risk factors (smoking, family history of CAD, hypertension, diabetes mellitus and hypercholesterolemia) were considered nominal variables. Initially, t test for continuous data and χ^2 ^for categorical data were used to assess the differences in socio-demographic and medical history factors between cases and controls. Genotype frequencies of E298D polymorphism were compared in the two groups of participants with Fisher's exact test. To study the univariate association of MI with the eNOS genotype we modeled the data through logistic regression analysis (crude odds ratios); the relevant p-values derived from Wald test for dominant and recessive models and Armitage test for trend for additive model. Subsequently, a multivariate analysis was performed to estimate the risk of MI for TT versus GT+GG after adjustment for a series of possible risk factors and confounders (adjusted odds ratios). Results were accepted as statistically significant when two-tailed p-values were less than 0.05. The SAS statistical package (Version 9.1, SAS Institute Inc, Cary, NC) was used in all analyses whereas power calculation was performed on QUANTO.

## Results

### Univariate analyses

Setting minor allele frequency (MAF) at 0.3, expected odds ratio from 1.5 to 2.5 (univariate odds ratio for the relationship between TT/GG+GT and the risk for MI), alpha 0.05 and power 80%, on QUANTO, mode of inheritance seems to result in an odds ratio of at least 2.3.

Table 1 shows the distribution of 204 cases with MI and 218 controls by socio-demographic and clinical characteristics. Regarding age and gender, no significant differences were identified between cases and controls (p = 0.580 and p = 0.658 respectively). The mean BMI was significantly higher in cases as compared to controls (p = 0.028). The prevalence of atherogenic risk factors such as smoking, family history of CAD, hypertension, diabetes mellitus and hypercholesterolemia was significantly higher in the patient group.

**Table 1:** Demographic and clinical characteristics of myocardial infarction (MI) among patients and controls

Variables	MI Patients (n = 204)	Controls (n = 218)	P-value*^†^*
Age (years)			0.580
< 60	104 (51.0)	117 (53.7)	
60+	100 (49.0)	101 (46.3)	
Gender			0.658
Male	178 (87.2)	187 (85.8)	
Female	26 (12.8)	31 (14.2)	
BMI (kg/m^2^)	26.88 ± 3.47	26.19 ± 2.92	0.028
Smoking			< 0.0001
No	37 (18.1)	99 (45.4)	
Yes	167 (81.9)	119 (54.6)	
Family history of CAD			< 0.0001
No	119 (58.3)	195 (89.5)	
Yes	85 (41.7)	23 (10.5)	
Hypertension			< 0.0001
No	59 (28.9)	167 (76.6)	
Yes	145 (71.1)	51 (23.4)	
Diabetes mellitus			< 0.0001
No	131 (64.2)	208 (95.4)	
Yes	73 (35.8)	10 (4.6)	
Hypercholesterolemia			< 0.0001
No	28 (13.7)	198 (90.8)	
Yes	176 (86.3)	20 (9.2)	

Data are presented as mean ± SD or N (%)

^†^P-values derived from t-test for continuous variables; chi-square test was used for categorical variables

Table 2 presents the distribution of genotypes GG, GT and TT in the two groups of participants. The frequencies of the eNOS GG genotype were 40.7% and 49.5%, of heterozygous GT 46.1% and 43.6% and of homozygous TT 13.2% and 6.9% among cases and controls respectively. This difference was statistically significant (p = 0.046). Concerning the allele frequencies of the E298 to D transition in the MI and control group were calculated at 36.3% and 28.7% respectively (p = 0.019).

**Table 2:** Genotype frequencies of E298D polymorphism in MI patients and controls

eNOS E298D Polymorphism	MI Patients N (%)	Controls N (%)	P-value*^†^*
eNOS			0.046
GG	83 (40.7)	108 (49.5)	
GT	94 (46.1)	95 (43.6)	
TT	27 (13.2)	15 (6.9)	
allele			0.019
G	260 (63.7)	311 (71.3)	
T	148 (36.3)	125 (28.7)	

^†^P-values derived from genotypic and allelic Fisher's exact tests

In the univariate analyses of the data (Table 3), TT homozygous, compared with the group of homozygous and heterozygous individuals GG+GT (recessive model), were at higher risk for MI (OR = 2.06, 95%CI = 1.06-4.00, p = 0.032). Even if TT homozygous were compared with E298 homozygous GG alone, they were still more likely to develop MI (OR = 2.34, 95%CI: 1.17-4.68, p = 0.016). We found no statistical significant evidence for a protective effect against the risk for MI among homozygous GG as compared to heterozygous GT (p = 0.220), whereas when comparing them to GT+TT (dominant model) the result was not significant (p = 0.068). According to the additive model, one allele increase was associated with 43% higher risk of MI (OR = 1.43, 95%CI: 1.07-1.93, p = 0.018) as compared to the baseline category of homozygous GG.

**Table 3:** Logistic regression-derived, crude odds ratios (ORs) and 95% confidence intervals (CIs) for the risk of myocardial infarction (MI) in carries of the 298D allele among the study participants

eNOS E298D	Crude analysis		
	
	ORs	95% CI	P-value
***Recessive model***			
TT vs GG+GT	2.06	1.06-4.00	0.032*
***Dominant model***			
GG vs GT+TT	0.70	0.48-1.03	0.068*
***Additive model***			
T allele vs GG	1.43	1.07-1.93	0.018**
***Other models***			
TT vs GG	2.34	1.17-4.68	0.016*
TT vs GT	1.82	0.91-3.64	0.090*
T allele vs G allele	1.42	1.06-1.89	0.022*
GG vs GT	0.78	0.52-1.16	0.220*

*P-values derived from Wald test

** P-value derived from Armitage test for trend

### Multivariate analyses

Based on the univariate findings suggesting an increased risk for TT homozygous and the idea that the recessive model of inheritance seems to be more appropriate than dominant or additive for the needs of such a research [23], we further performed multivariate analyses in order to examine the relation of MI with a series of available parameters. Due to the presence of mutual confounding, correlation or interaction, it was not possible to examine all the study covariates simultaneously. Several alternative statistical models are performed and presented in Table 4. In all these models, homozygous TT as compared to GT+GG were found to be at statistically significant higher risk for developing MI with the estimated odds ratios ranging from 2.04 to 2.89. Gender does not seem to be associated with the risk for MI, whereas the effect of age is unclear. There was some evidence that increasing BMI increases the risk for MI, but the results were at the borderline of statistical significance. Lastly, strong evidence was found for statistically significant, positive association of MI with smoking, family history of CAD and hypertension. The small cell frequencies of diabetes mellitus and hypercholesterolemia did not allow us to include these two variables in the multivariate analyses.

**Table 4:** Logistic regression-derived odds ratios (ORs) and 95% confidence intervals (95% CIs) for the risk of myocardial infarction (MI) by eNOS E298D, controlling for demographic and medical history variables among the 422 study participants

Variable	Category or increment	**ORs**^**1 **^**(95% CIs)**	**ORs**^**2 **^**(95% CIs)**	**ORs**^**3 **^**(95% CIs)**	**ORs**^**4 **^**(95% CIs)**	**ORs**^**5 **^**(95% CIs)**
		p-value	p-value	p-value	p-value	p-value
eNOS E298D	TT vs. GT+GG	2.06 (1.06-4.00)	2.04 (1.05-3.96)	2.41 (1.18-4.92)	2.57 (1.18-5.60)	2.89 (1.11-7.51)
		0.032	0.036	0.016	0.018	0.029
Age	60+ vs. < 60 years		1.11 (0.75-1.65)	1.76 (1.13-2.75)	2.24 (1.38-3.64)	1.00 (0.54-1.80)
			0.602	0.013	0.001	0.969
Gender	female vs. male		0.86 (0.48-1.53)	1.26 (0.67-2.37)	1.19 (0.62-2.30)	1.37 (0.66-2.87)
			0.606	0.470	0.608	0.401
BMI	2 kg/m^2 ^more			1.14 (1.00-1.30)	1.13 (0.98-1.30)	1.12 (0.97-1.30)
				0.057	0.096	0.132
Smoking	yes vs. no			4.93 (2.98-8.15)	5.53 (3.21-9.54)	6.83 (3.64-12.84)
				< 0.0001	< 0.0001	< 0.0001
Family history of CAD	yes vs. no				6.97 (3.98-12.21)	6.91 (3.69-12.94)
					< 0.0001	< 0.0001
Hypertension	yes vs. no					11.91 (6.70-21.17)
						< 0.0001

1 = Model 1: Unadjusted odds ratio for eNOS E298D

2 = Model 2: Odds ratio for mutually adjusted eNOS E298D, age and gender

3 = Model 3: Odds ratio for mutually adjusted eNOS E298D, age, gender, BMI and smoking

4 = Model 4: Odds ratio for mutually adjusted eNOS E298D, age, gender, BMI, smoking and family history of CAD

5 = Model 5: Odds ratio for mutually adjusted eNOS E298D, age, gender, BMI, smoking, family history of CAD and hypertension

## Discussion

We performed a case-control study of the common E298D polymorphism of the eNOS gene. Similarly to recent studies [8-11,13,17-20], we found a significant association between homozygous carriers of the T allele and the occurrence of MI in the Greek population. The risk of developing MI was found to be about two-fold higher for 298D (homozygous carriers of the T allele) as compared with individuals carrying the wild type E298 allele, either heterozygous (GT) or homozygous (GG) (p = 0.032). Lack of an increased risk of MI in the eNOS GT heterozygous suggests that the risk of MI posed by the eNOS T allele is not dominantly expressed and that the increased risk is confined to eNOS TT homozygous. This finding confirms previous presumptions of a recessive gene effect. Patients who are homozygous for the E298D polymorphism are genetically predisposed to MI (Table 3). The results of the present study are in accordance with the findings of a recent meta-analysis of the Glu298Asp polymorphism, including 14 studies with 6036 IHD cases and 6106 controls. According to this meta-analysis, the summary OR under a fixed-effect model showed that individuals homozygous for the Asp298 allele were 1.31 times more likely to develop ischemic heart disease (95% CI, 1.13 to 1.51; p = 0.0003) whereas the dominant model showed a non-significant association between carriers of ≥1 Asp298 allele and the risk of ischemic heart disease (OR, 1.06; 95% CI, 0.97 to 1.15; *p = *0.21) [23].

MI is a multifactorial disease. Further genetic and environmental risk factors, such as age, gender, BMI, smoking habits and family history as well as accompanying disorders like high blood pressure and hypercholesterolemia contribute significantly to the susceptibility of MI.

The homozygous T allele carriers, as suggested by the univariate analysis of the present study, have an increased risk to develop MI. We performed a multivariate analysis to examine the additive influence of age, gender, BMI, smoking habits and family history to the TT genotype. The increased risk of the TT gene remains even if we add the variables smoking, hypertension and family history. BMI seems to tentatively elevate MI risk, while gender seems to be unrelated to MI. Data from a recent study confirmed that predominantly younger T allele carriers of the eNOS E298D gene polymorphism with various coronary high-risk profiles have an increased risk to suffer MI [11]. Taking into account that the cardiovascular disease is a multicausal process and since other than genetic risk factors develop in the course of life, genetic factors are more likely to affect young rather than old people and may contribute to many different mechanisms leading to atherosclerotic lesions [24,25]. Nevertheless, our study does not confirm those findings since the role of age remained unclear.

The association between eNOS E298D polymorphism and MI is strongly affected by cigarette smoking. In a previous study it was demonstrated that the variant 298D allele is particularly associated with significantly lower endothelial-dependent vasodilation in smokers, with no significant effect in non-smokers [26]. We observed that the combination of smoking and 298D allele further increased OR for MI, suggesting that there is a gene-environment interaction. Smokers, eNOS TT homozygous have greater risk of MI than eNOS GT heterozygous and GG homozygous smokers. These data, possibly suggest, that presence of established underlying endothelial dysfunction, as observed among cigarette smokers, may be necessary for this polymorphism to attenuate endothelial function and predispose patients to increased cardiovascular risk. The impact of smoking, however, in females could not be demonstrated. It has been reported that the development of endothelial dysfunction and atherosclerosis in premenopausal females may follow a different pattern than in men, probably due to estrogen-mediated protection of the endothelium [26].

The results of the present study strongly suggest that eNOS gene may be a risk factor for MI. Although the mechanism by which E298D polymorphism confers susceptibility to MI is not clear, several studies have described a possible role of NO in the pathogenesis of MI and suggest that endothelial NO may have an important atheroprotective role beyond its effect on vessel tone and blood pressure [27,28]. This study suggests that an alteration in the activity of the vascular NO system and the decreased amount of endothelial NO, due to the E298D mutation, may promote atherosclerosis and thrombosis that lead to MI. However, the relation of the E298D mutation to the severity of coronary atherosclerosis has not been proven yet [8]. Impaired effects of NO on the cardiovascular system lead to dysregulation of vascular tone, platelet aggregation and leukocyte adhesion and smooth muscle cell proliferation, all of which promote coronary atherosclerosis and thrombosis [9].

Two previous studies showed a significant association between the 298D allele and the risk of MI in the Greek population [18,19]. A third study by Andrikopoulos et al [20] failed to demonstrate such an association.

This study adds to the gradually developing consensus that E298D polymorphism of the eNOS gene seems to be associated with MI occurrence. It is possible that TT is closely linked to the etiology of MI even after adjusting for known MI risk factors. The underlying mechanisms are still speculative but it appears that further studies are needed to explore whether E298D polymorphism is an independent risk factor or an indirect marker of different genetic and environmental factors.

## Study Limitations

No case control study is immune to criticism concerning case and control selection and the present one is no exception. However, the identification in the present study of most established risk factors for MI indicates that possible selection and information biases were not important enough to challenge the validity of the results. We have no reason to believe that cases were not representative of people suffering MI in the country. However, because of the overall low frequency of TT (15 subjects with TT genotype in the control group and 27 in the case group), the results should be viewed with caution. Notably, given the expected low frequency of TT in the general population, this type of studies face practical difficulties in achieving high number of participants. Further studies could provide an insight into the role of E298D polymorphism of the eNOS gene in the MI occurrence.

## Conclusions

This study indicates that E298D polymorphism of the eNOS gene seems to be associated with MI occurrence in the Greek population. We found evidence that homozygous TT is positively related to the risk of MI and this association is independent of possible effects of other known MI risk factors. Smokers, hypertensive and those with a family history of CAD are more likely to develop MI.

## Competing interests

The authors declare that they have no competing interests.

## Authors' contributions

CD carried out the molecular genetic analyses, participated in the design of the study and helped to draft the manuscript. ND conceived the study, participated in the design and coordination of the study and drafted the final manuscript. OL carried out the design of primers and probes. MR and DP helped with the statistical analysis and to draft the manuscript. DVC participated in its design, coordinated the recruitment of the patients and helped to draft the manuscript. All authors read and approved the final manuscript.

## Pre-publication history

The pre-publication history for this paper can be accessed here:

http://www.biomedcentral.com/1471-2350/11/133/prepub
